# A PEDOT:PSS/MXene-based actuator with self-powered sensing function by incorporating a photo-thermoelectric generator†

**DOI:** 10.1039/d3ra06290b

**Published:** 2023-11-08

**Authors:** Yongqiang Qian, Peidi Zhou, Yi Wang, Ying Zheng, Zhiling Luo, Luzhuo Chen

**Affiliations:** a Fujian Provincial Key Laboratory of Quantum Manipulation and New Energy Materials, College of Physics and Energy, Fujian Normal University Fuzhou 350117 China ChenLZ@fjnu.edu.cn; b Institute of Smart Marine and Engineering, Fujian University of Technology Fuzhou 350118 China; c Fujian Provincial Collaborative Innovation Center for Advanced High-Field Superconducting Materials and Engineering Fuzhou 350117 China; d Fujian Provincial Engineering Technology Research Center of Solar Energy Conversion and Energy Storage Fuzhou 350117 China; e Department of Obstetrics, Fuzhou Second Hospital Fuzhou 350007 China yingzhengfzhosp@163.com

## Abstract

Actuators with sensing functions are becoming increasingly important in the field of soft robotics. However, most of the actuators are lack of self-powered sensing ability, which limits their applications. Here, we report a light-driven actuator with self-powered sensing function, which is designed to incorporate a photo-thermoelectric generator into the actuator based on poly(3,4-ethylenedioxythiophene):poly(styrenesulfonate) (PEDOT:PSS)/MXene composite and polyimide. The actuator shows a large bending curvature of 1.8 cm^−1^ under near-infrared light (800 mW cm^−2^) irradiation for 10 s, which is attribute to photothermal expansion mismatch between PEDOT:PSS/MXene composite and polyimide. Simultaneously, the actuator shows enhanced thermoelectric properties with Seebeck coefficient of 35.7 μV K^−1^, which are mainly attributed to a combination of energy filtering effects between the PEDOT:PSS and MXene interfaces as well as the synergistic effect of its charge carrier migration. The output voltage of the actuator changes in accordance with the bending curvature, so as to achieve the self-powered sensing function and monitor the operating state of the actuator. Moreover, a bionic flower is fabricated, which not only simulates the blooming and closing of the flower, but also perceives the real-time actuation status through the output voltage signal. Finally, a smart Braille system is elaborately designed, which can not only simulate Braille characters for tactile recognition of the blind people, but also automatically output the voltage signal of Braille for self-powered sensing, enabling multi-channel output and conversion of light energy. This research proposes a new idea for exploring multifunctional actuators, integrated devices and self-powered soft robots.

## Abstract 2

A light-driven actuator with self-powered sensing function is proposed, incorporating a photo-thermoelectric generator into the actuator based on PEDOT:PSS/MXene composites.

## Graphical abstract

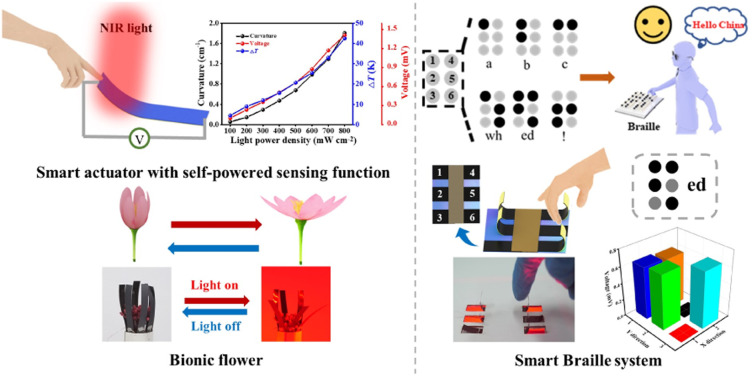

## Introduction

Actuators enable the conversion of external energy stimuli into mechanical energy, and they have great potential applications in soft robotics,^1–4^ biomedicine,^5,6^ and electronic skin.^7,8^ However, people are no longer satisfied with the simple mechanical deformation of actuators with the development of intelligent technology. Actuators have gradually become multi-functional devices combining signal transmission, strain sensing, feedback, control, and data analysis, which will complicate the design and fabrication of the integrated device. Furthermore, most of the reported actuators with sensing functions are mostly integrated with resistive or capacitive strain sensors, which monitor the shape-deformation amplitudes of the actuator by investigating the change in resistance or capacitance of the actuator.^9–12^ Consequently, they all require additional electrical power, which will also significantly degrade the portability of the device and increase energy losses.^13–16^

Up to now, a great deal of research has been performed to find sustainable and small-scale energy harvesting systems to actuate sensing devices. Self-powered devices can convert environmental energy (mechanical, chemical, thermal energy, *etc.*) into electrical energy.^17^ For example, triboelectric nanogenerators (TENG)^18–20^ and piezoelectric nanogenerators (PENG)^21–23^ that convert small or low-frequency mechanical energy into electrical energy are considered promising technologies for self-powered sensing and energy harvesting. The self-powered devices are widely used in applications such as medical monitoring,^24,25^ self-powered sensing,^22,26^ and human–machine interaction.^27–29^ Nevertheless, long-term mechanical impingement and extrusion deformation will significantly degrade the durability of these self-powered devices.^30,31^ At the same time, high sensitivity is essential for actuators with sensing functions. However, most multi-functional self-powered actuators based on TENG and PENG are usually a combination of several devices, and the interference between devices can severely degrade measurement accuracy. Consequently, there is benefit for developing a highly integrated actuator to solve the above problems.

Self-powered devices integrated with thermoelectric (TE) materials are attractive for smart devices with sensing functions due to their capability to generate electricity without direct contact and their sensitive perception of temperature. The principle of electricity generation of TE materials is based on the Seebeck effect.^32^ Due to the temperature gradient, charge carriers (electrons or holes) within a solid material migrate, which in turn forms an electrical potential difference between two ends of the material. The TE generator converts low-quality waste heat from the environment into electrical energy by utilizing TE materials. Conventional inorganic TE materials, such as the bismuth–tellurium–antimony–selenium (Bi–Te–Sb–Se) alloy family, have been characterized by their high TE properties.^33^ However, they normally have poor mechanical durability due to their brittleness, which partly limits their applications.^34–36^ More importantly, with the development of smart technology, multi-functional devices with portable, flexible and self-powered fascinating features are becoming increasingly attractive. Therefore, it is required to develop a simple and effective method to fabricate flexible self-powered sensing actuators with compact structures.

Conductive polymers^37^ and MXene^38,39^ have triggered increasing research interest in the field of self-powered devices due to their easy-to-prepare, scalable and flexible fascinating properties. In particular, poly(3,4-ethylenedioxythiophene):poly(styrenesulfonate) (PEDOT:PSS) has good conductivity, high transparency and easy processability,^40–44^ while Ti_3_C_2_T_*x*_ has excellent photothermal conversion properties.^45–48^ In recent years, they have become one of the hot topics in the field of flexible self-powered devices, but there is still huge room for improvement. Firstly, the mechanical and self-powered properties of the actuators can be enhanced by further studying the structural design of the materials and utilizing the complementarity of material properties. Secondly, self-powered actuators with more compact structures can be fabricated using the similarity of device structures. Finally, if the self-powered sensing function enables the actuator to be used as a sensor to monitor the motion of the actuator in real-time, the device will be more multi-functional.

**Fig. 1:**
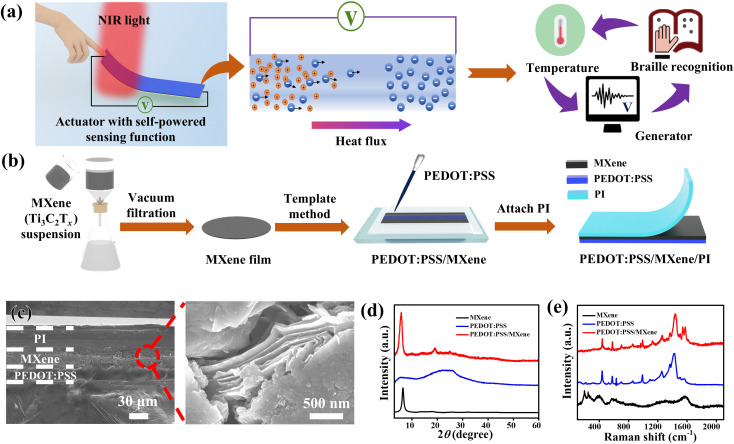
(a) Schematic diagram of the mechanism and application of the actuator with self-powered sensing function. (b) Schematic diagram of the fabrication process of PEDOT:PSS/MXene/PI film. (c) Cross-sectional SEM image of PEDOT:PSS/MXene/PI film, and the high magnification image showing the typical layered structure of MXene. (d) XRD patterns of MXene, PEDOT:PSS, and PEDOT:PSS/MXene. (e) Raman spectra of MXene, PEDOT:PSS, and PEDOT:PSS/MXene.

Herein, we report a light-driven actuator with self-powered sensing function based on PEDOT:PSS, MXene (Ti_3_C_2_T_*x*_), and polyimide (PI) composite. The highlight of our research is incorporating a photo-thermoelectric (PTE) generator into the actuator for self-powered sensing, while achieving large shape deformations. The multi-layered PEDOT:PSS/MXene/PI actuator shows a bending actuation with a curvature of 1.8 cm^−1^ when illuminated by near-infrared (NIR) light. The actuation is mainly attributed to the difference in volume variation between PI and PEDOT:PSS/MXene.^42,48,49^ Meanwhile, an output voltage signal is obtained at the two ends of the actuator along with the bending deformation of the actuator. Fig. 1(a) shows a schematic diagram of a PTE generator incorporated into actuator for the self-powered sensing function, which is due to the photothermal and TE properties of the PEDOT:PSS/MXene composite. When one end of the PEDOT:PSS/MXene composite is irradiated by NIR light, the temperature rises, resulting in a temperature gradient at the two ends. Due to the TE effect,^50–53^ holes inside PEDOT:PSS flow from the hot end to the cold end while electrons inside MXene flow from the hot end to the cold end. Moreover, the PEDOT:PSS/MXene composite film shows an enhanced Seebeck coefficient due to the energy filtering effect caused by the difference in the work functions of PEDOT:PSS and MXene.^54–56^ As a result, the PEDOT:PSS/MXene/PI composite outputs a voltage signal at both ends. The output voltage change is in step with the bending curvature change of the actuator. Importantly, the voltage signal can be used to monitor the operating state of the actuator without requiring an additional electrical power. Furthermore, we demonstrate two self-powered sensing systems, including a bionic flower and a smart Braille system. When the bionic flower is illuminated by NIR light, it can not only simulate the movement of plants, but also monitor the actuation status of the actuator in real-time by outputting voltage signals. The smart Braille system can not only simulate Braille for tactile recognition and temperature sensing of the blind, but also output voltage signals. In the future, the system can even be combined with a computer to convert voltage signals into sound signals, which enables the simultaneous multi-channel output and conversion of light energy. We hope this study can provide a good inspiration for future exploration of multi-functional integrated self-powered devices.

## Results and discussions

### Fabrication and characterization of PEDOT:PSS/MXene/PI film

Fig. 1(b) shows a schematic diagram of the fabrication process of the PEDOT:PSS/MXene/PI film. First, a suspension of the pretreated MXene (Ti_3_C_2_T_*x*_) was added to deionized water. A self-supporting MXene film was obtained by vacuum filtration and natural drying. Subsequently, the PEDOT:PSS dispersion was added dropwise to the surface of the MXene film, where the weight ratio of PEDOT:PSS to MXene was 1 : 4, and the reason for choosing this ratio will be explained later. Since there are electrostatic interactions between the negatively charged MXene layers^57^ and the positively charged PEDOT:PSS,^58^ the PEDOT:PSS/MXene bilayer can be obtained without any gluing. Optical photographs of the prepared PEDOT:PSS/MXene film are shown in Fig. S1 (ESI†). It can be seen that the PEDOT:PSS/MXene film can be bent and folded, which shows its flexibility. Finally, a PI film was attached *in situ* on the PEDOT:PSS/MXene film to form the PEDOT:PSS/MXene/PI composite. More experimental details are described in the Experimental section. Scanning electron microscope (SEM) images of two surfaces of PEDOT:PSS/MXene are shown in Fig. S2 (ESI†). They show the smooth surface of PEDOT:PSS and the two-dimensional wrinkles of the MXene. Fig. 1(c) shows a cross-sectional SEM image of the PEDOT:PSS/MXene/PI film, in which the high magnification image shows the typical layered structure of MXene (right panel of Fig. 1(c)).^45,59^ The PEDOT:PSS/MXene film is intimately combined to PI film with a total thickness of 81 μm, where the PEDOT:PSS/MXene film has a thickness of 46 μm. The X-ray diffraction (XRD) patterns of MXene, PEDOT:PSS, and PEDOT:PSS/MXene are shown in Fig. 1(d). The MXene shows a sharp (002) characteristic peak (6.4°) compared to Ti_3_AlC_2_ (MAX phase) (Fig. S3, ESI†), which shows that MXene was successfully etched.^60^ The PEDOT:PSS shows wide peaks at 5.9° and 25.8°, respectively, without characteristic peaks.^61^ The PEDOT:PSS/MXene composite shows a trend of superposition of MXene and PEDOT:PSS peaks, and reveals a sharp characteristic peak of (002). Fig. 1(e) and S4 (ESI†) show the Raman spectra of MXene, PEDOT:PSS, and PEDOT:PSS/MXene. The MXene shows two characteristic Raman bands at 198 cm^−1^ and 390 cm^−1^ respectively. The PEDOT:PSS shows the corresponding characteristic peaks for the weak C_α_–C_β_ inter-ring stretching (1253 cm^−1^), the single C_α_–C_β_ stretch (1366 cm^−1^), the strong C_α_=C_β_ symmetric stretch (1425 cm^−1^) and the C_α_=C_β_ antisymmetric stretch (1564 cm^−1^).^62^ Furthermore, the characteristic peak of the PEDOT:PSS/MXene film is shifted to 1438 cm^−1^ compared to the characteristic peak of the original PEDOT:PSS (1425 cm^−1^). This phenomenon demonstrates that interactions occur at the interface layer between PEDOT:PSS and MXene, which allows PEDOT:PSS to be tightly bound to MXene.^63^

### TE properties of PEDOT:PSS/MXene film

**Fig. 2:**
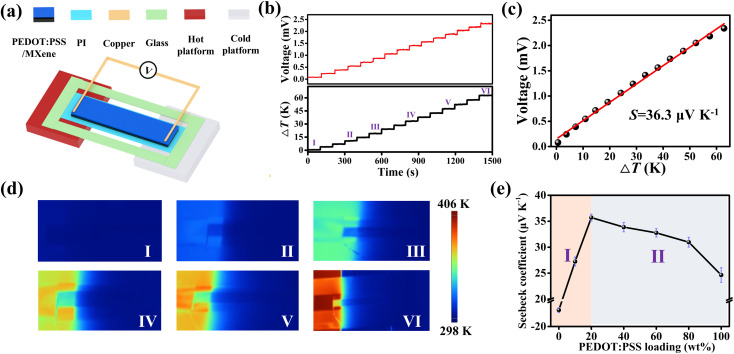
(a) Schematic diagram of the measurement device for TE property based on PEDOT:PSS/MXene film. (b) Output voltage and Δ*T* of the PEDOT:PSS/MXene film at different stages during heating by a hot platform. (c) Output voltage of the PEDOT:PSS/MXene film as a function of Δ*T*. (d) Infrared images of the PEDOT:PSS/MXene film at different stages during the heating by a hot platform. (e) Seebeck coefficient of the PEDOT:PSS/MXene film as a function of the loading percentage of the PEDOT:PSS.

Fig. 2(a) shows a schematic diagram of testing the TE properties of PEDOT:PSS/MXene film. Notably, the PI film is non-conductive and does not have thermoelectric properties. Therefore, the PI film is used as a substrate for holding the PEDOT:PSS/MXene film during the TE property test. Moreover, a hot platform was used as the heat source to create a spatial temperature gradient between the two ends of the PEDOT:PSS/MXene film. During the experiments, copper foil electrodes were connected to the hot and cold ends of the PEDOT:PSS/MXene film. At the same time, the PEDOT:PSS/MXene film was fixed in a glass frame by the PI film to avoid bending and movement of the film during the heating. It is worth noting that one end of the PEDOT:PSS/MXene film (1 cm) was placed on the hot platform and the rest of the film was left suspended in the room temperature platform during the test. More details are described in the Experimental section and Fig. S5 (ESI†). One end of the PEDOT:PSS/MXene film is heated by constantly changing the temperature of the hot platform, while the temperature of the other end remains almost unchanged. The temperature distributions of PEDOT:PSS/MXene film in the beginning and after 60 min of heating are shown in Fig. S6 (ESI†). As a result, a spatial temperature difference (Δ*T*) is generated at the two ends of the PEDOT:PSS/MXene film. Due to the TE effect, a voltage signal will be obtained at the two ends. Fig. 2(b) shows Δ*T* and its corresponding open-circuit voltage (*V*_oc_). It can be seen that the *V*_oc_ changes synchronously with the Δ*T*. The *V*_oc_ is up to 2.3 mV with Δ*T* of 63 K. The Seebeck coefficient (*S*) for the PEDOT:PSS/MXene film is obtained by the equation *S* = *V*_oc_/Δ*T*, which is approximately 36.3 μV K^−1^, as shown in Fig. 2(c). The corresponding infrared images at different stages are shown in Fig. 2(d). It can be seen that the Δ*T* between the two ends of the PEDOT:PSS/MXene film gradually increases as the temperature of the hot platform continues to rise. It is worth noting that the enhancement of the Seebeck coefficient is significant in enhancing the performance of TE devices. Therefore, the effect of the content of PEDOT:PSS in PEDOT:PSS/MXene film on the Seebeck coefficient was studied, as shown in Fig. 2(e). It was found that there was a negative TE effect with a Seebeck coefficient of −17.2 μV K^−1^ for pure MXene film without PEDOT:PSS, which is consistent with previous reports.^64^ The variation of Δ*T* and output voltages of pure MXene film are shown in Fig. S7 (ESI†).

Interestingly, the Seebeck coefficient of PEDOT:PSS/MXene films firstly increases with the increase of PEDOT:PSS content (zone I) and then gradually decreases (zone II). The Seebeck coefficient of the PEDOT:PSS/MXene film increases significantly to a maximum of 36.3 μV K^−1^, while the content of coated PEDOT:PSS is below 20 wt%. The Δ*T* and output voltages of the PEDOT:PSS/MXene film with 10 wt% PEDOT:PSS are shown in Fig. S8 (ESI†). The Seebeck coefficient of the PEDOT:PSS/MXene film starts to decrease when the content of coated PEDOT:PSS is higher than 20 wt%. The details are described in Fig. S9–S11 (ESI†). With content of 100 wt% (*i.e.* pure PEDOT:PSS), the Seebeck coefficient was only 24.6 μV K^−1^, as shown in Fig. S12 (ESI†). In zone I, the Seebeck coefficient of PEDOT:PSS/MXene film significantly increases with the increasing PEDOT:PSS content and even shows a shift from negative to positive values until reaching a maximum value. This enhancement of Seebeck coefficient is mainly related to the energy filtering effects between the interfacial layers of PEDOT:PSS and MXene as well as the superposition of PEDOT:PSS and MXene's self-charge carrier migration.^65–69^

Since the work function of PEDOT:PSS (4.84 eV)^56,70,71^ is larger than that of MXene (4.61 eV),^55^ the Fermi energy level of PEDOT:PSS is lower than that of MXene.^56^ Therefore, there will be a potential barrier layer between PEDOT:PSS and MXene, which will selectively allow higher energy charge carriers to pass through and scatter lower energy charge carriers. After the layer screening, the average energy of the carriers is raised, which in turn causes the enhancement of the Seebeck coefficient.^72–77^ In addition, due to the negative TE property of MXene, electrons can migrate from the hot end to the cold end. On the contrary, the PEDOT:PSS has positive TE properties and holes can migrate from the hot end to the cold end, which also accumulates more charge carriers at both ends of the material. In summary, under the same Δ*T*, the PEDOT:PSS/MXene film will have a larger potential difference between the two ends. In zone II, the Seebeck coefficient of the PEDOT:PSS/MXene film will show a gradually decreasing trend with the increase of the PEDOT:PSS content. This phenomenon may be due to the increase of PEDOT:PSS content, which leads to the thickness of PEDOT:PSS larger than that of MXene. At that time, the charge carrier migration inside the PEDOT:PSS/MXene composite film is gradually dominated by the self-carrier migration of PEDOT:PSS. With the loading of PEDOT:PSS increased to approach 100 wt%, the Seebeck coefficient of the composite film is almost comparable to that of pure PEDOT:PSS. Significantly, the experimental samples used for subsequent PTE and actuation tests were all made of PEDOT:PSS/MXene composites with the PEDOT:PSS and MXene weight ratio of 1 : 4.

### PTE properties of PEDOT:PSS/MXene film

**Fig. 3:**
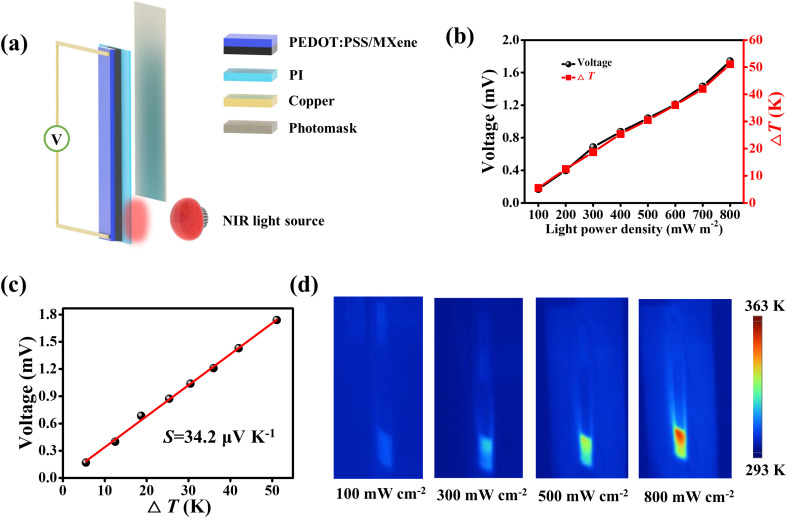
(a) Schematic diagram of measurement for PTE property of PEDOT:PSS/MXene film. (b) Output voltage and Δ*T* of PEDOT:PSS/MXene film as a function of light power density. (c) Output voltage of PEDOT:PSS/MXene film as a function of Δ*T*. (d) Infrared images of PEDOT:PSS/MXene film under different light powers.

As excellent photothermal conversion materials, MXene and PEDOT:PSS are widely used in photothermal actuators^78–80^ and PTE generators.^63,70^ Here, NIR light was used as the heat source to investigate the PTE properties of PEDOT:PSS/MXene film. Fig. 3(a) shows a schematic diagram of the device for the PTE performance tests. The PEDOT:PSS/MXene film was held vertically in a glass frame with a PI film. Similarly, the PI film is used as a substrate to fix the PEDOT:PSS/MXene film. Electrodes fabricated by copper foil were embedded in two ends of the PEDOT:PSS/MXene film. During the test, NIR light was irradiated at one end of the PEDOT:PSS/MXene film (1 cm), while the surrounding area was shielded with a photomask that was made of copper foil coated with PI film. More details are described in the Experimental section and Fig. S13 (ESI†). Owing to the photothermal effect, when one end of the PEDOT:PSS/MXene film was irradiated with NIR light, the temperature increased while the temperature at the other end remained almost unchanged. In accordance with the TE effect, an output voltage signal will be detectable at both ends of the composite film. Fig. S14 (ESI†) shows the Δ*T* and output voltage of the PEDOT:PSS/MXene film under different light powers. With the increase of light power, there is a positive correlation between Δ*T* and the output voltage of the PEDOT:PSS/MXene film. When the light power density was up to 800 mW cm^−2^, the Δ*T* was 51.1 K and the output voltage was 1.74 mV. The corresponding maximum Δ*T* and open-circuit voltage under different light powers are displayed in Fig. 3(b). As shown in Fig. 3(c), the Seebeck coefficient was obtained to be 34.2 μV K^−1^. Fig. 3(d) shows the infrared images of the PEDOT:PSS/MXene film under different light powers. It can be seen that the temperature of the irradiated part of the composite film increases with the increasing of the light power density.

### Actuation and PTE properties of PEDOT:PSS/MXene/PI film

**Fig. 4:**
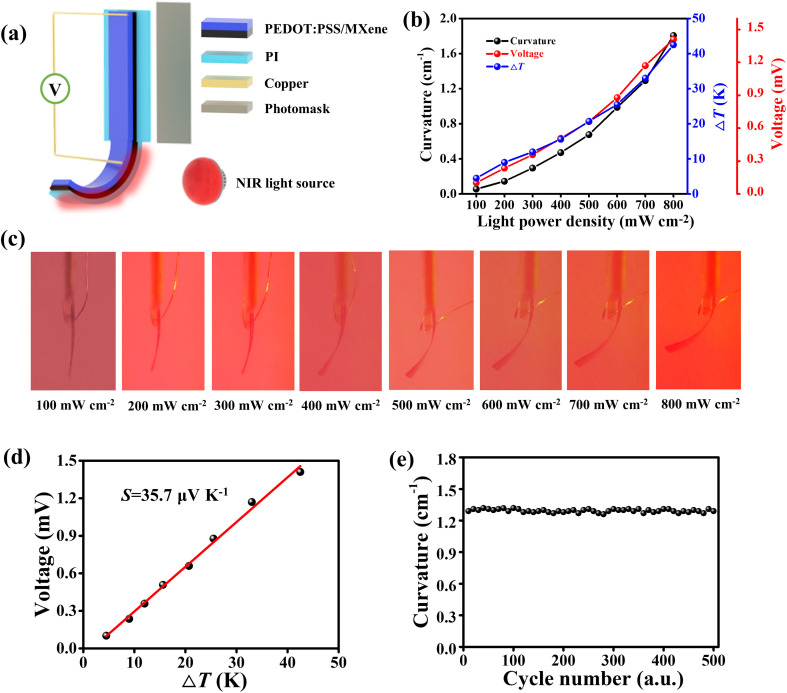
(a) Schematic diagram of the actuation and PTE property measurement for PEDOT:PSS/MXene/PI actuator. (b) Bending curvature, Δ*T*, and the maximum output voltage of the PEDOT:PSS/MXene/PI actuator as a function of light power density. (c) Optical photographs of the shape deformation of PEDOT:PSS/MXene/PI actuator under different light powers. (d) Output voltage of the PEDOT:PSS/MXene/PI actuator as a function of Δ*T*. (e) Repeatability test on the actuation performance of PEDOT:PSS/MXene/PI actuator (700 mW cm^−2^ for 500 cycles).

It is valuable to fabricate more integrated and multi-functional actuators based on devices of similar structure. The PEDOT:PSS/MXene/PI film has a multilayer structure and good PTE properties. Meanwhile, the photothermal actuator also possesses a layered structure, of which the shape deformation is mainly attributed to the volume change mismatch between the layers. The PI has a positive coefficient of thermal expansion.^49^ The PEDOT:PSS combined with some hydrophilic materials has a negative coefficient of thermal expansion.^42^ The hydrophilic MXene has no significant thermal expansion.^48^ Based on these properties, a light-driven actuator with PTE properties was fabricated based on PEDOT:PSS/MXene/PI films. Fig. 4(a) shows a schematic diagram of the actuator incorporated with a PTE generator. The PEDOT:PSS/MXene/PI film (length of 4.5 cm) was fixed in a glass frame with the remaining part suspended (2 cm). The electrodes were embedded into the top and bottom ends (1.5 cm) respectively. More details are depicted in the Experimental section and Fig. S15 (ESI†).

Due to the PTE effect, when one end of the actuator was irradiated by NIR light, the temperature increased. At the same time, the temperature at the other end almost remained unchanged. An output voltage can be generated at the two ends of the actuator. Meanwhile, the actuator bent towards the PEDOT:PSS/MXene side due to the thermal expansion of the PI film and water-loss shrinkage of the PEDOT:PSS/MXene composite.^42,63^ With the increasing of light power, the Δ*T* and output voltage change in the same trend, as shown in Fig. S16 (ESI†). Fig. 4(b) shows the maximum Δ*T*, output voltage, and bending curvature of the actuator under different light powers. As the light power density increased to 800 mW cm^−2^, the Δ*T* was 42.5 K, the output voltage was 1.41 mV and the bending curvature was 1.8 cm^−1^. The calculation of the actuator curvature is described in Note S1 (ESI†) and Fig. S17 (ESI†). The optical photographs of the actuator under different light powers are shown in Fig. 4(c). The results show that the bending curvature of the actuator gradually increased with the increasing of light power. Fig. 4(d) shows the output voltage of the actuator increased with Δ*T*, and the Seebeck coefficient was calculated to be 35.7 μV K^−1^.

Finally, we performed a cyclic test of the PEDOT:PSS/MXene/PI actuator under the light power density of 700 mW cm^−2^ for 500 cycles, and the Δ*T* and output voltage were recorded simultaneously. As shown in Fig. S18 (ESI†), the Δ*T* and output voltage of the actuator can remain stable for a long period of time. The magnified image shows that there was no significant degradation during the entire cyclic test. Fig. 4(e) shows the maximum bending curvatures of the PEDOT:PSS/MXene/PI actuator during 500 cycles, which were stable at ∼1.3 cm^−1^, indicating its good durability. Fig. S19 (ESI†) shows that the Seebeck coefficient also has good cycling stability, which is very important for long-term usage. As a comparative test, the actuation and PTE properties of the actuator based on PEDOT:PSS/PI without MXene were recorded, as shown in Fig. S20 (ESI†). Fig. S20(a) (ESI†) shows a schematic of the PEDOT:PSS/PI actuator. The dimensions of the PEDOT:PSS/PI actuator is shown in Fig. S20(b) (ESI†). A similar process of construction and testing of the actuator was performed compared to the testing of the PTE properties of PEDOT:PSS/MXene/PI actuators. Fig. S20(c) (ESI†) exhibits the optical photographs of the bending deformation of the PEDOT:PSS/PI actuator under different light powers. As the light power increases, the Δ*T*, output voltage of the actuator changes in a consistent trend, as shown in Fig. S20(d) (ESI†). Under the light power density of 800 mW cm^−2^, the output voltage of the PEDOT:PSS/PI actuator was only 1.01 mV. At the same time, the maximum bending curvature was only 0.84 cm^−1^ and the Seebeck coefficient was 24.1 μV K^−1^ (Fig. S20(e), ESI†). Furthermore, the bending curvature of the PEDOT:PSS/MXene/PI actuator and the output voltage have a good linear relationship, as shown in Fig. S21 (ESI†). In summary, on the one hand, the PEDOT:PSS/MXene/PI actuator shows an enhanced Seebeck coefficient, which is attributed to the energy filtration at the PEDOT:PSS/MXene interface and the accumulation of self-charged carrier migration. On the other hand, the additional MXene enhances the photothermal properties of the composite film, resulting in a larger bending deformation of the actuator.

### Application of PTE generators with sensing function

#### Bionic flower

**Fig. 5:**
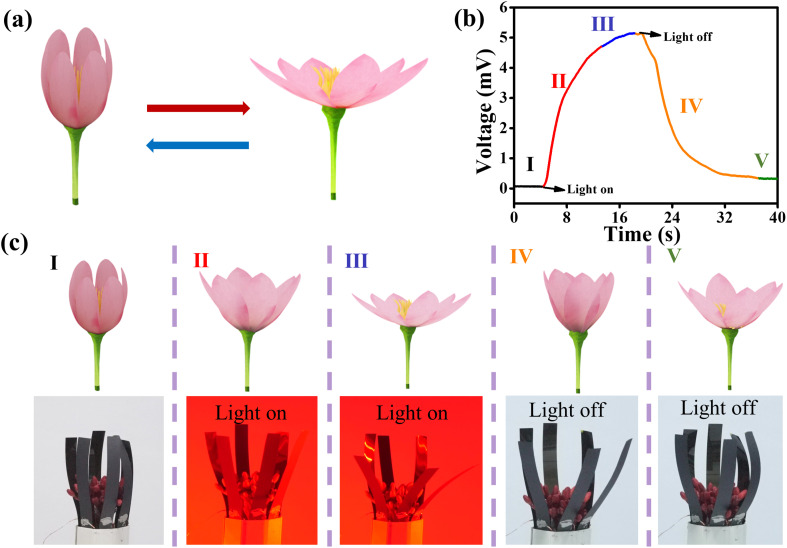
(a) Schematic diagram of the blooming and closing of a bionic flower. (b) The voltage signals of bionic flower in different states. (c) Optical photographs of bionic flowers, corresponding to different states in (b).

We designed a bionic flower based on PEDOT:PSS/MXene/PI actuators, which consisted of six actuators connected in series. As the bending curvature of the actuator has a very good linear relationship with the output voltage, the self-powered voltage signal can be used to monitor the movement behavior of the bionic flower, such as the blooming and closing of flowers. The experimental details about the bionic flower are depicted in Fig. S22 (ESI†). Fig. 5(a) is a schematic diagram showing the blooming and closing of the bionic flower. When the NIR light was turned on (700 mW cm^−2^), the bionic flower gradually bloomed and the output voltage continued to rise (red line in Fig. 5(b)). When the bionic flower fully bloomed, the output voltage rose to the maximum value (blue line in Fig. 5(b)). When the NIR light was turned off, the flower gradually closed and the output voltage started to drop (gold line in Fig. 5(b)). When the bionic flower was closed, the output voltage remained stable (green line in Fig. 5(b)). The above process is demonstrated in Movie S1 (ESI†). Furthermore, Fig. 5(c) shows the optical photographs of the bending state of the bionic flower. The results show that the different states of the flower can be sensed in real-time using the self-powered voltage signal.

#### Smart Braille system

**Fig. 6:**
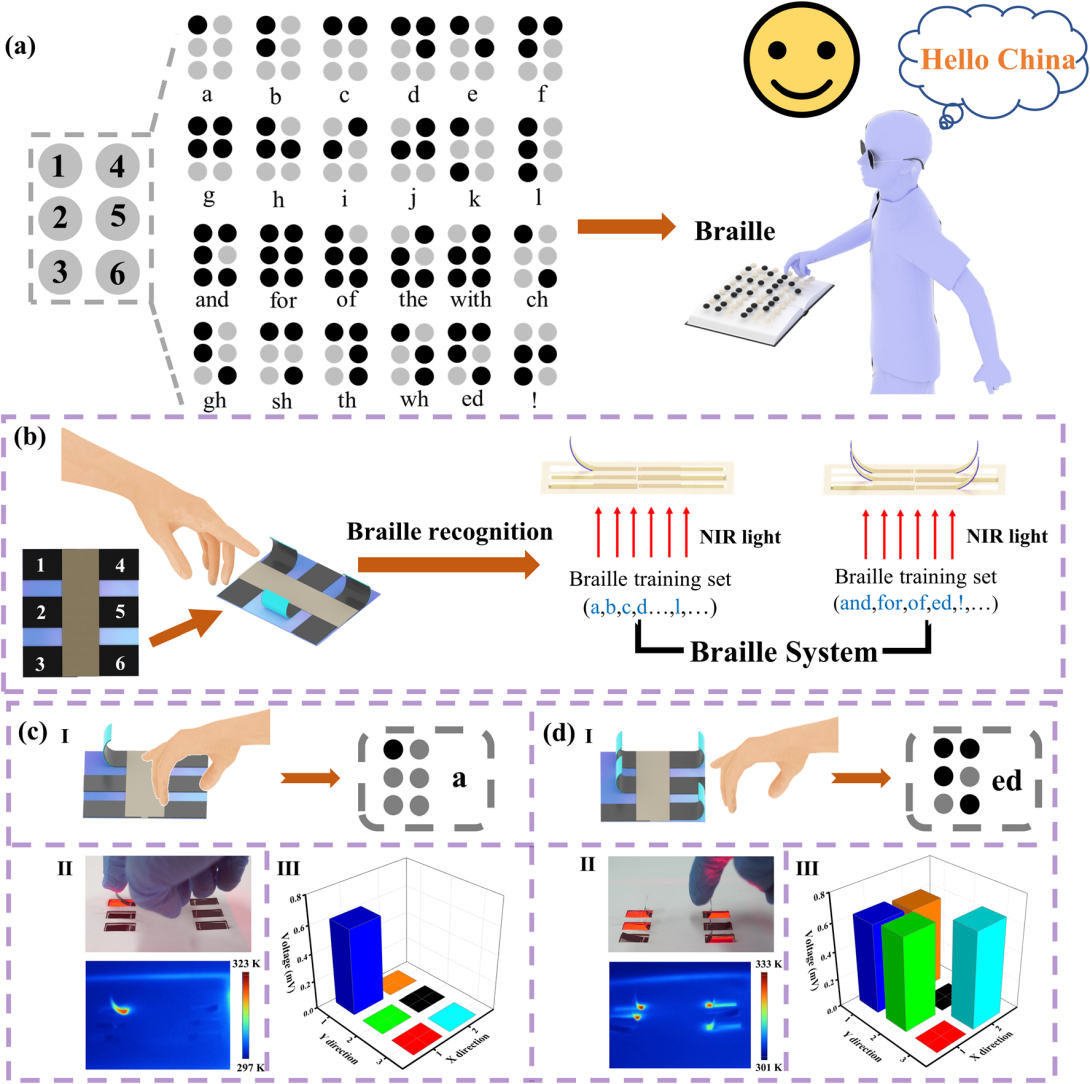
(a) A list of the meanings of different Braille characters and Braille recognition for the blind people. (b) Schematic diagram of the smart Braille system. (c) Demonstration of a simple Braille character “a” based on PEDOT:PSS/MXene/PI actuators. (d) Demonstration of a complex Braille character “ed” based on the PEDOT:PSS/MXene/PI actuators.

The emergence of Braille was a major advancement for people with limited vision, enabling them to read and write independently. The principle of Braille is based on the sense of touch, and blind people need to perceive Braille through their fingers, as shown in Fig. 6(a). The Braille characters consist of 1–6 sites, which represent different meanings by raising different sites, such as letters and symbols. Compared to traditional writing, blind people can have access to information and understand the world through Braille. Inspired by Braille, we elaborated a smart Braille system that simulates and monitors Braille recognition for the blind people. The example of the smart Braille system is presented here with six actuators instead of the six sites of Braille. When different actuators were irradiated by NIR light, the actuators were bent to resemble raised Braille. Therefore, Braille letters and characters can be simulated by arranging combinations of different positions of the actuators (Fig. 6(b)). Fig. 6(c(I)) shows a schematic diagram of Braille character “a” and the corresponding Braille. When the actuator at site 1 was irradiated by NIR light (700 mW cm^−2^), this actuator bent, while the actuators at the other sites remained unchanged. The above process is demonstrated in Movie S2 (ESI†). When the device was touched by hand, the bumps at the corresponding sites can be sensed, which is similar to the recognition of Braille for the blind people. At the same time, an optical photograph and an infrared image of Braille character “a” were recorded, as shown in Fig. 6(c(II)). It is noted that the designation of the actuator to be illuminated by NIR light is achieved by controlling the light plate on the back of the actuator, as shown in Fig. S23(a) (ESI†). Due to the PTE effect, an output voltage signal was monitored at the actuator on site 1. And the output voltage changed synchronously with the bending of the actuator. Fig. 6(c(III)) shows the output voltages of the six actuators, and schematic diagrams of the front and back for the Braille device are shown in Fig. S23(b) (ESI†).

Similarly, a schematic of the Braille “ed” and the corresponding Braille are shown in Fig. 6(d(I)). When the actuators at sites 1, 2, 4 and 6 were irradiated, these actuators bent while the actuators at the other sites remained unchanged. The above process is demonstrated in Movie S3 (ESI†). An optical photograph of the actuator and the corresponding infrared image are shown in Fig. 6(d(II)). Due to the PTE effect, output voltages were generated at the actuators on 1, 2, 4, and 6 sites. The output voltage signal matches the bending state of the corresponding actuators, as shown in Fig. 6(d(III)). The front and back sides of the Braille device for “ed” are shown in Fig. S23(c) (ESI†). The real-time voltage signal variation for different Braille symbols were recorded (Fig. S24, ESI†). The experimental results show that the smart Braille system can not only simulate tactile recognition of Braille for the blind people, but also monitor the type of Braille utilizing the corresponding voltage signal of the system. In addition, Braille recognition is difficult for normal people to understand in real life, due to lack of appropriate training. It is valuable that this system can record the Braille information output by self-powered voltage signal of the actuators. In the future, this electronic signal can be converted into sound output for anyone to recognize Braille. The above two application examples fully demonstrate that the self-powered actuator based on PEDOT:PSS/MXene/PI composite can not only mimic the bending deformations of plants and smart devices, but also monitor the shape deformation of the actuator utilizing the output voltage signal.

## Conclusions

In summary, we propose a flexible light-driven actuator with self-powered sensing function. The flexible self-supporting film based on the PEDOT:PSS/MXene composite was fabricated by the combination of vacuum filtration and template method. On the one hand, the PEDOT:PSS/MXene composite film shows an enhanced Seebeck coefficient due to the combination of the energy filtering effect between the PEDOT:PSS and MXene interfaces and the synergistic effect of its self-charge carrier migration. When the content of PEDOT:PSS in PEDOT:PSS/MXene composite is 20 wt%, the composite film reaches the maximum Seebeck coefficient. On the other hand, the PEDOT:PSS/MXene/PI actuator shows good actuation performance under NIR light irradiation due to the difference in volume expansion between the PEDOT:PSS/MXene composite and PI films. The actuator shows a maximum bending curvature of 1.8 cm^−1^ under NIR light irradiation of 800 mW cm^−2^ for 10 s. More importantly, the actuator with incorporated PTE generator can monitor the bending deformation of the actuator in real-time using the spontaneously generated voltage signal instead of additional applied power. As application examples, we designed a bionic flower and a smart Braille system. Both of these self-powered sensing systems can monitor the operating status of the actuator using voltage signals, thus enabling multi-channel output and conversion of light energy. The actuators with self-powered sensing function have a wide range of applications in human–computer interaction, remote monitoring, and self-powered robots.

## Experimental section

### Materials

The PEDOT:PSS was purchased as a suspension containing 1 wt% solids from Zhuhai Kaiwei Optoelectronics Technology Co., Ltd. The LiF was purchased from Shanghai Maclean Biochemistry Co., Ltd. The Ti_3_AlC_2_ (MAX) was purchased from 11 Technology Co., Ltd. HCl and dimethyl sulfoxide (DMSO) were purchased from Sinopharm Chemical Reagent Co., Ltd. The PI film was purchased from an online supermarket. Deionized water was used for all the water in the whole experiment.

### Synthesis of MXene (Ti_3_C_2_T_*x*_) suspensions

MXene (Ti_3_C_2_T_*x*_) was synthesized by etching Ti_3_AlC_2_ (MAX phase) with a mixture of LiF and HCl. Specifically, LiF (2.3 g) was added to a mixture of concentrated hydrochloric acid (25 mL, 12 mol L^−1^) and deionized water (5 mL). Subsequently, Ti_3_AlC_2_ (1 g) was added to this mixture and magnetically stirred for 10 min. The above mixture was then placed in a hydrothermal kettle and heated in water at 80 °C for 72 h. Afterwards, the obtained suspension was centrifuged at 4500 rpm min^−1^ and then washed with hydrochloric acid (100 mL, 0.1 mol L^−1^) and deionized water (100 mL) until the supernatant reached neutrality. The Ti_3_C_2_T_*x*_ was obtained by being dried at 60 °C for 12 h. Afterwards, DMSO was used for intercalation of Ti_3_C_2_. Specifically, pretreated Ti_3_C_2_T_*x*_ (500 mg) powder was added to DMSO (30 mL), magnetically stirred at 25 °C for 18 h, and then centrifuged at high speed (10 000 rpm min^−1^) for 15 min. The resulting precipitate was added to deionized water (75 mL) and sonicated for 3 h to obtain a MXene suspension (6.67 mg mL^−1^).

### Fabrication of PEDOT:PSS/MXene/PI film

The self-supporting MXene film was prepared by vacuum filtration of the MXene suspension. Specifically, a suspension of MXene (15 mL) was added to deionized water and stirred. Then, the aqueous solution was poured into a funnel and filtered to remove the deionized water. The MXene film was left to dry in a natural environment. Afterwards, the MXene film was cut into long strips with dimensions of 2 cm × 6 cm. The MXene film was fixed to the glass plate with double-sided tape. PEDOT:PSS (1 mL) was then applied evenly in drops to the surface of the MXene film. The PEDOT:PSS/MXene film was obtained by being dried under natural conditions for 12 h. The PI film was attached to the PEDOT:PSS/MXene film. Finally, the PEDOT:PSS/MXene/PI film with a multilayer structure was obtained.

### Measurement of the TE properties of PEDOT:PSS/MXene film

First, the PI film with dimensions of 7 cm × 2 cm was attached to a PEDOT:PSS/MXene film with dimensions of 5.5 cm × 1 cm (Fig. S5, ESI†). Then, the PEDOT:PSS/MXene/PI film was fixed in the glass frame. The copper foil electrodes were embedded in each end of the PEDOT:PSS/MXene/PI film by silver glue, Fig. 2(a) shows the exact position of the electrode embedding. During the TE performance test, one end (1 cm) of the PEDOT:PSS/MXene/PI film was placed on the hot platform, the other end was kept horizontal and the middle part was left suspended. The device is held in place by the PI film to prevent the PEDOT:PSS/MXene/PI film from bending or moving during heating. The temperature and output voltage at each end of the PEDOT:PSS/MXene/PI film were recorded while the hot platform was operating. It is worth noting that since the cold end of the PEDOT:PSS/MXene/PI film is not heated and the change in temperature is extremely small, it is assumed that it does not change. Therefore, the same operation was done in the later test sessions.

### Measurement of the PTE properties of PEDOT:PSS/MXene film

First, the PEDOT:PSS/MXene film is fixed in the glass frame, referring to the section on testing of TE properties for details of the method and dimensions. Copper foil electrodes are then embedded in the two ends of the PEDOT:PSS/MXene/PI film. For the PTE performance test, the PEDOT:PSS/MXene/PI film is placed vertically with a photomask blocking the top of it (4.5 cm). When the NIR light is on, only the bottom of the actuator (1 cm) can be irradiated. The temperature and output voltage at the two ends of the PEDOT:PSS/MXene/PI film are recorded simultaneously.

### Measurement of the actuation and PTE properties of PEDOT:PSS/MXene/PI film

First, a PEDOT:PSS/MXene film with dimensions of 6.5 cm × 1 cm was fixed in the glass frame, referring to the section on TE performance testing for details of the method and dimensions. The copper foil electrodes were embedded and Fig. S15 (ESI†) shows the position of the electrodes. During the performance of actuation and self-powered sensing testing, the PEDOT:PSS/MXene/PI film was placed vertically with a light baffle blocking it above (4.5 cm). The bottom of the actuator (2 cm) can be irradiated by the NIR light, as shown in Fig. S15 (ESI†). The temperature and output voltage at the two ends of the PEDOT:PSS/MXene/PI film were recorded simultaneously. The bending deformation of the PEDOT:PSS/MXene/PI film was recorded with a digital camera. It is necessary to note that this method is also applicable to the actuation and self-powered sensing performance tests of the PEDOT:PSS/PI film, as shown in Fig. S20 (ESI†).

### Characterizations

The SEM images of the material were taken by field emission scanning electron microscopy (Hitachi SU8010). A Raman spectrometer (HORIBA JobinYvon Evolution) with a 532 nm He–Ne laser line was used to record the Raman spectra. An X-ray diffractometer (Rigaku MiniFlex II) with Cu Kα radiation (*λ* = 0.15405 nm) was used to record X-ray diffraction (XRD). An infrared thermal imager (Fluke Ti10) was used to capture infrared images of the samples. A digital source meter (Keithley 2410) was used to record output electrical signals. A laser-sighted infrared thermometer (Optris LS) was used to record the sample temperature. A digital camera (Sony ILCE 6000) was used to record optical photographs and videos.

## Conflicts of interest

There are no conflicts to declare.

## Supplementary Material

RA-013-D3RA06290B-s001

RA-013-D3RA06290B-s002

RA-013-D3RA06290B-s003

RA-013-D3RA06290B-s004
