# Supplementary material for: A PEDOT:PSS/MXene-based actuator with self-powered sensing function by incorporating a photo-thermoelectric generator
Source: RSC Adv. 2023 Nov 8;13(46):32722–33. doi: 10.1039/d3ra06290b (PMC10630741; doi:10.1039/d3ra06290b)
Supplement: RA-013-D3RA06290B-s004 [file RA-013-D3RA06290B-s004.pdf]

Electronic Supplementary Material (ESI) for RSC Advances.
This journal is © The Royal Society of Chemistry 2023

Supporting Information

A PEDOT:PSS/MXene-based actuator with self-powered sensing function by incorporating a photo-thermoelectric generator

Yongqiang Qian,‡[a,c,d] Peidi Zhou,‡[b] Yi Wang,[a,c,d] Ying Zheng,*[e] Zhiling Luo[a,c,d] and Luzhuo Chen *[a,c,d]

[a] Fujian Provincial Key Laboratory of Quantum Manipulation and New Energy Materials, College of Physics and Energy, Fujian Normal University, Fuzhou, 350117, China.

E-mail: ChenLZ@fjnu.edu.cn

[b] Institute of Smart Marine and Engineering, Fujian University of Technology, Fuzhou, 350118, China

[c] Fujian Provincial Collaborative Innovation Center for Advanced High-Field Superconducting Materials and Engineering, Fuzhou, 350117, China.

[d] Fujian Provincial Engineering Technology Research Center of Solar Energy Conversion and Energy Storage, Fuzhou, 350117, China.

[e] Department of Obstetrics, Fuzhou Second Hospital, Fuzhou, 350007, China

E-mail: yingzhengfzhosp@163.com

‡ These authors contributed equally to this work.

Supporting Figures

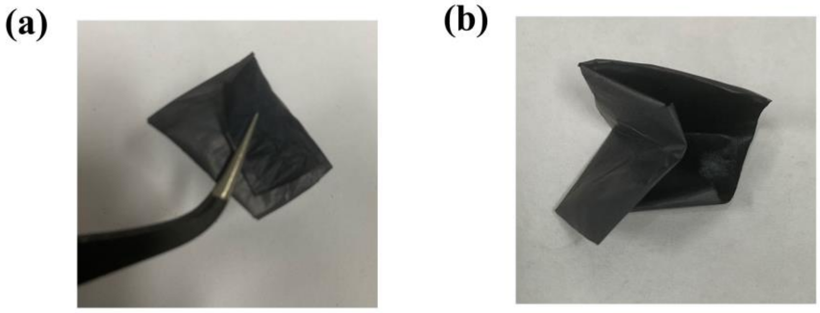

Fig. S1. (a) Optical photograph showing the bending of PEDOT:PSS/MXene film. (b) Optical photograph showing the folding of PEDOT:PSS/MXene film.

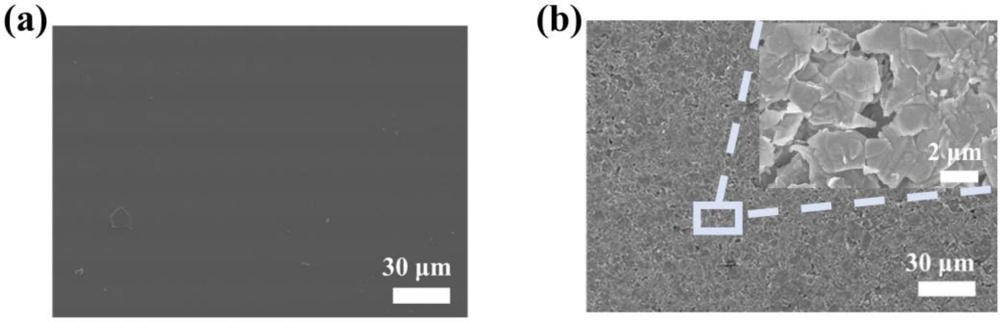

Fig. S2. (a) Surface SEM image of the PEDOT:PSS sides of the PEDOT:PSS/MXene film. (b) Surface SEM image of the MXene side of the PEDOT:PSS/MXene film.

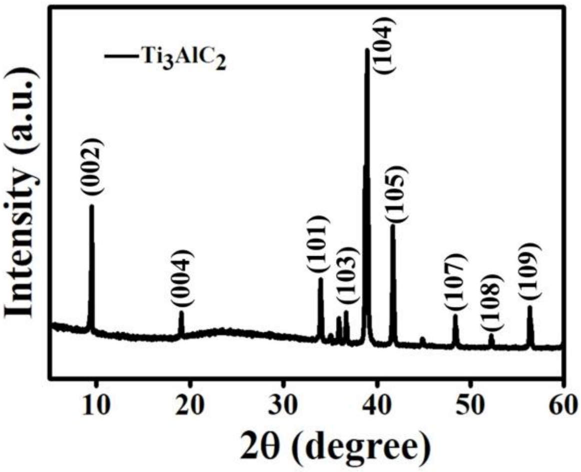

Fig. S3. XRD pattern of Ti_3AlC_2.

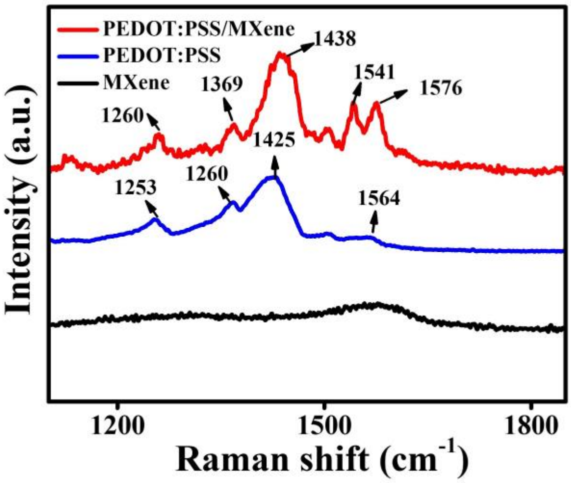

Fig. S4. Raman spectra of MXene, PEDOT:PSS and PEDOT:PSS/MXene in the 1100-1850 cm^{-1}.

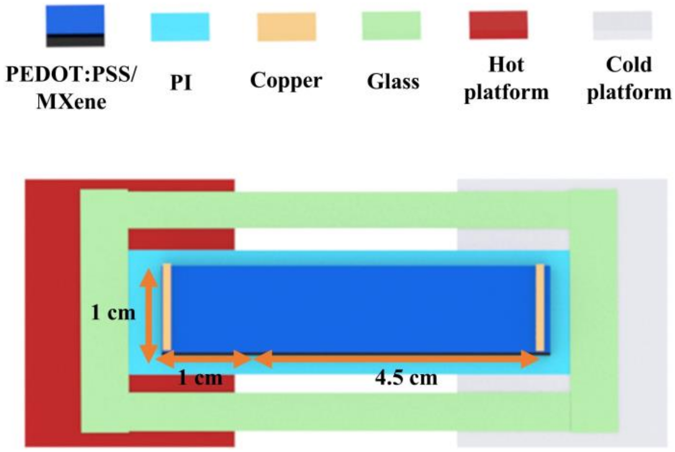

Fig. S5. Dimensions of PEDOT:PSS/MXene/PI film on a hot platform.

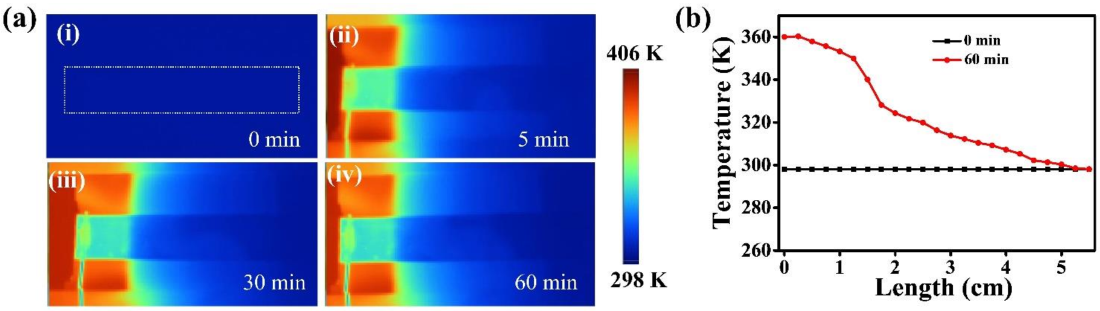

Fig. S6. (a) Infrared images of PEDOT:PSS/MXene film at (i) beginning, (ii) after 5 min, (iii) after 30 min and (iv) after 60 min of heating. (b) Temperature distributions of the PEDOT:PSS/MXene film in the beginning (0 min) and after 60 min of heating.

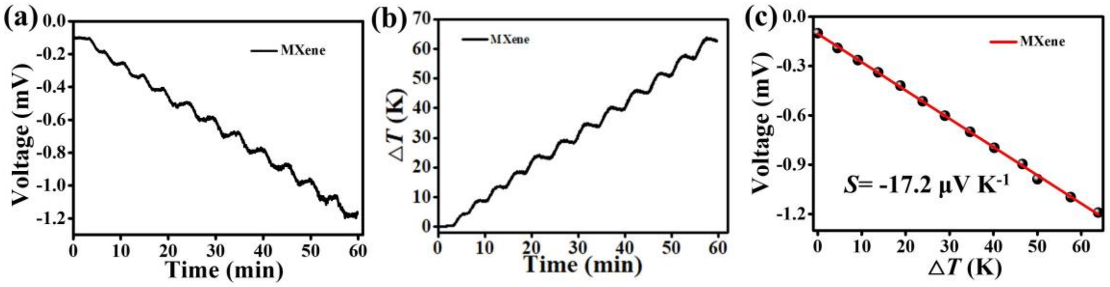

Fig. S7. (a) Output voltage based on MXene film during hot platform heating. (b) ΔT based on MXene film during hot platform heating. (c) Seebeck coefficient based on MXene film during hot platform heating.

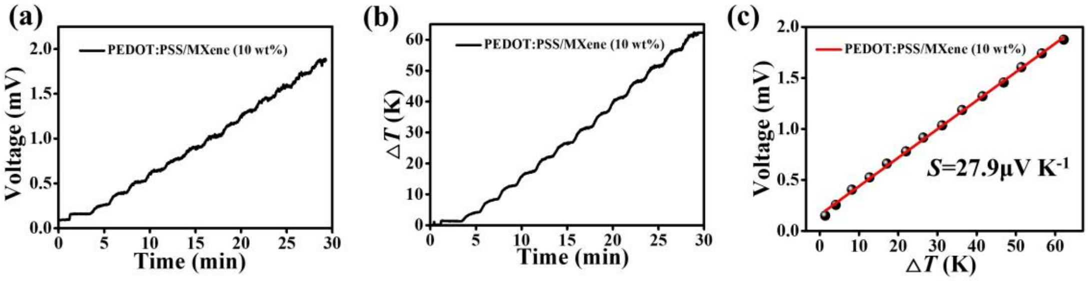

Fig. S8. (a) Output voltage of PEDOT:PSS/MXene film with PEDOT:PSS (10 wt%) during hot platform heating. (b) ΔT of PEDOT:PSS/MXene film with PEDOT:PSS (10 wt%) during hot platform heating. (c) Seebeck coefficient of PEDOT:PSS/MXene film with PEDOT:PSS (10 wt%) during hot platform heating.

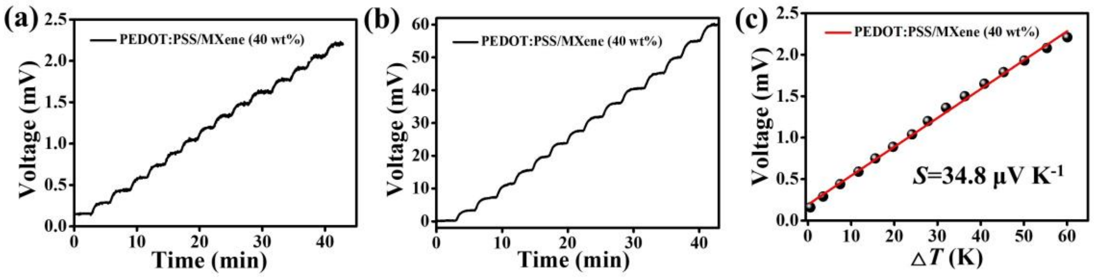

Fig. S9. (a) Output voltage of PEDOT:PSS/MXene film with PEDOT:PSS (40 wt%) during hot platform heating. (b) Δ*T* of PEDOT:PSS/MXene film with PEDOT:PSS (40 wt%) during hot platform heating. (c) Seebeck coefficient of PEDOT:PSS/MXene film with PEDOT:PSS (40 wt%) during hot platform heating.

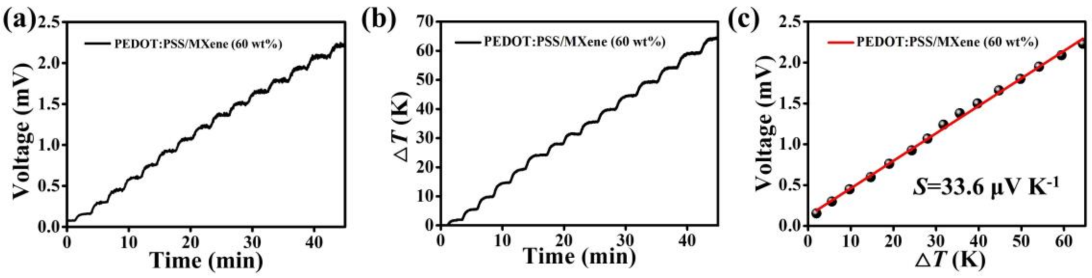

Fig. S10. (a) Output voltage of PEDOT:PSS/MXene film with PEDOT:PSS (60 wt%) during hot platform heating. (b) Δ*T* of PEDOT:PSS/MXene film with PEDOT:PSS (60 wt%) during hot platform heating. (c) Seebeck coefficient of PEDOT:PSS/MXene film with PEDOT:PSS (60 wt%) during hot platform heating.

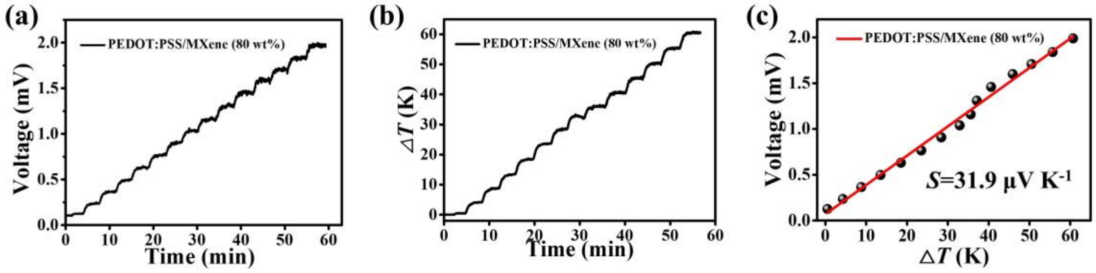

Fig. S11. (a) Output voltage of PEDOT:PSS/MXene film with PEDOT:PSS (80 wt%) during hot platform heating. (b) ΔT of PEDOT:PSS/MXene film with PEDOT:PSS (80 wt%) during hot platform heating. (c) Seebeck coefficient of PEDOT:PSS/MXene film with PEDOT:PSS (80 wt%) during hot platform heating.

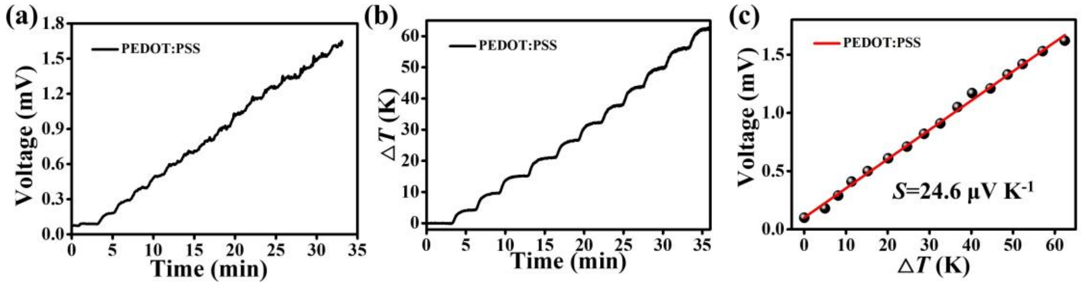

Fig. S12. (a) Output voltage of pure PEDOT:PSS film during hot platform heating. (b) ΔT of pure PEDOT:PSS film during hot platform heating. (c) Seebeck coefficient of pure PEDOT:PSS film during hot platform heating.

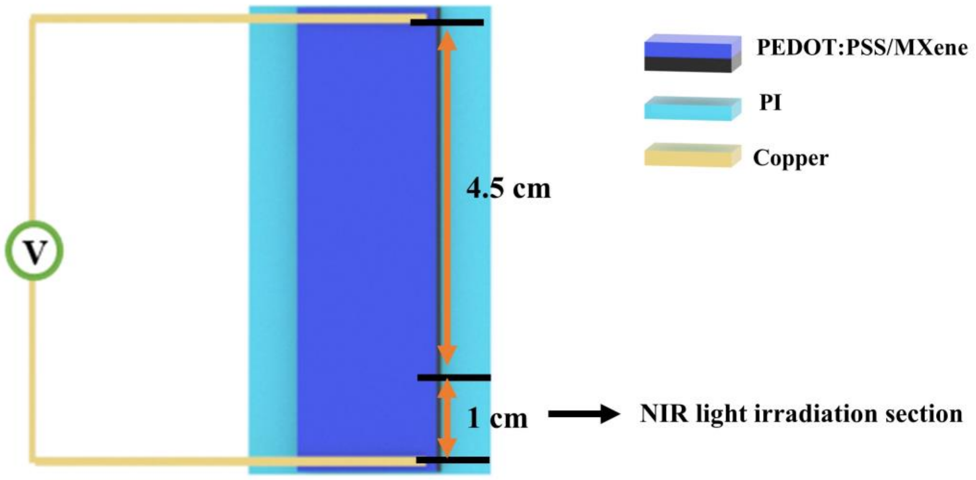

Fig. S13. Dimensions of PEDOT:PSS/MXene/PI films for PTE property test.

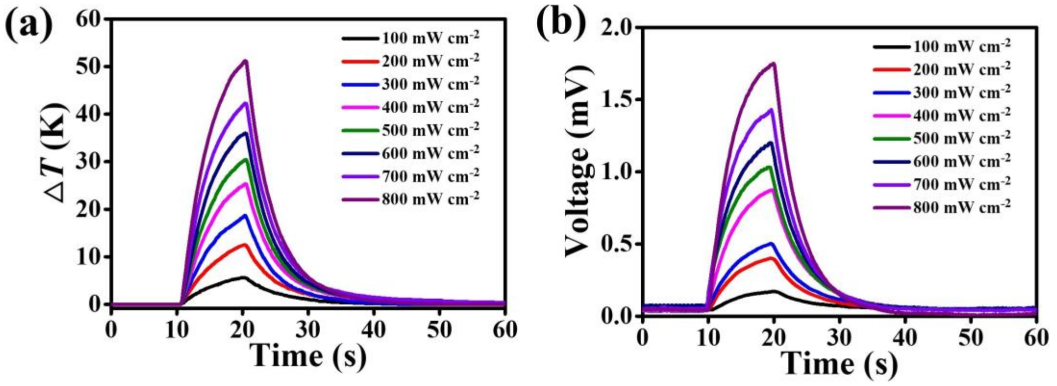

Fig. S14. PTE properties of the PEDOT:PSS/MXene film. (a) ΔT of the PEDOT:PSS/MXene film as a function of time under different light powers. (b) Output voltage of the PEDOT:PSS/MXene film as a function of time under different light powers.

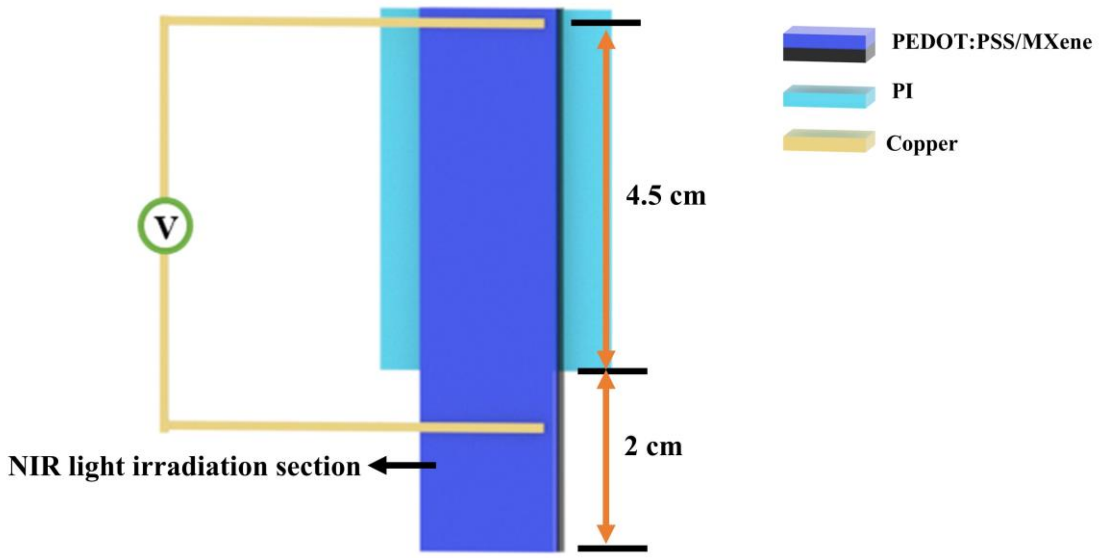

Fig. S15. Dimensions of the actuator based on PEDOT:PSS/MXene/PI for PTE and actuation properties test.

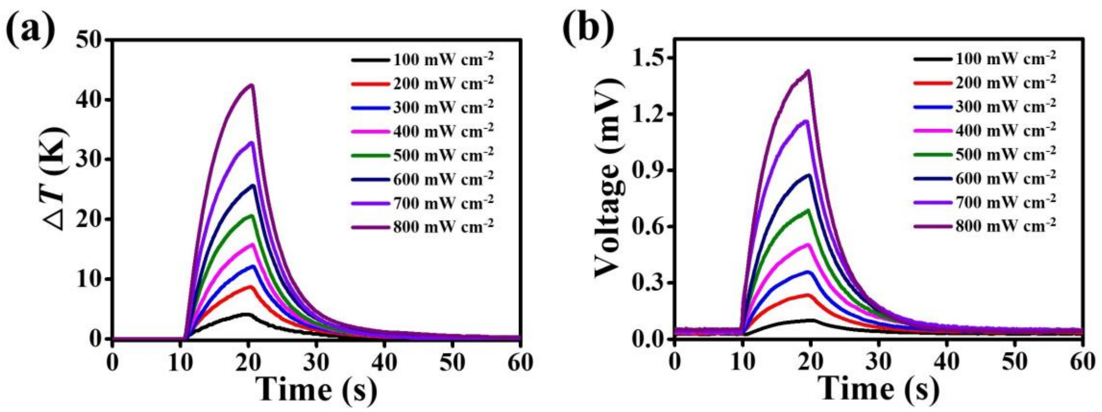

Fig. S16. PTE properties of the PEDOT:PSS/MXene/PI actuator during the actuation process. (a) ΔT of the PEDOT:PSS/MXene/PI actuator as a function of time under different light powers. (b) Output voltage of the PEDOT:PSS/MXene/PI actuator as a function of time under different light powers.

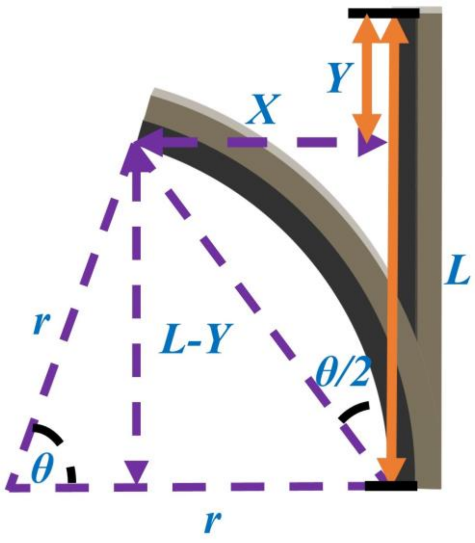

Fig. S17. Schematic diagram of the relevant parameters for curvature calculation.

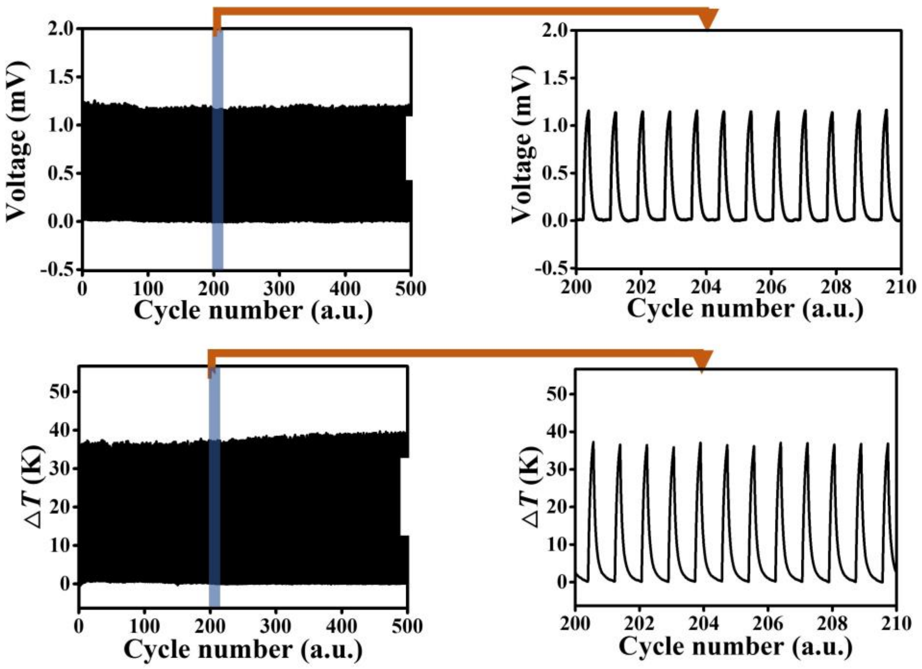

Fig. S18. (a) Output voltage of the PEDOT:PSS/MXene/PI actutor under light power density of 700 mW cm^{-2} during 500 cycles. (b) ΔT of the PEDOT:PSS/MXene/PI actuator under light power density of 700 mW cm^{-2} during 500 cycles.

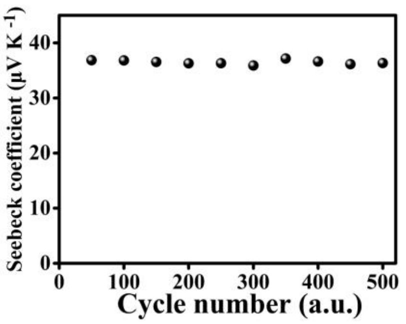

Fig. S19. Seebeck coefficient of the PEDOT:PSS/MXene/PI actuator during 500 cycles under light power density of 700 mW cm^{-2}.

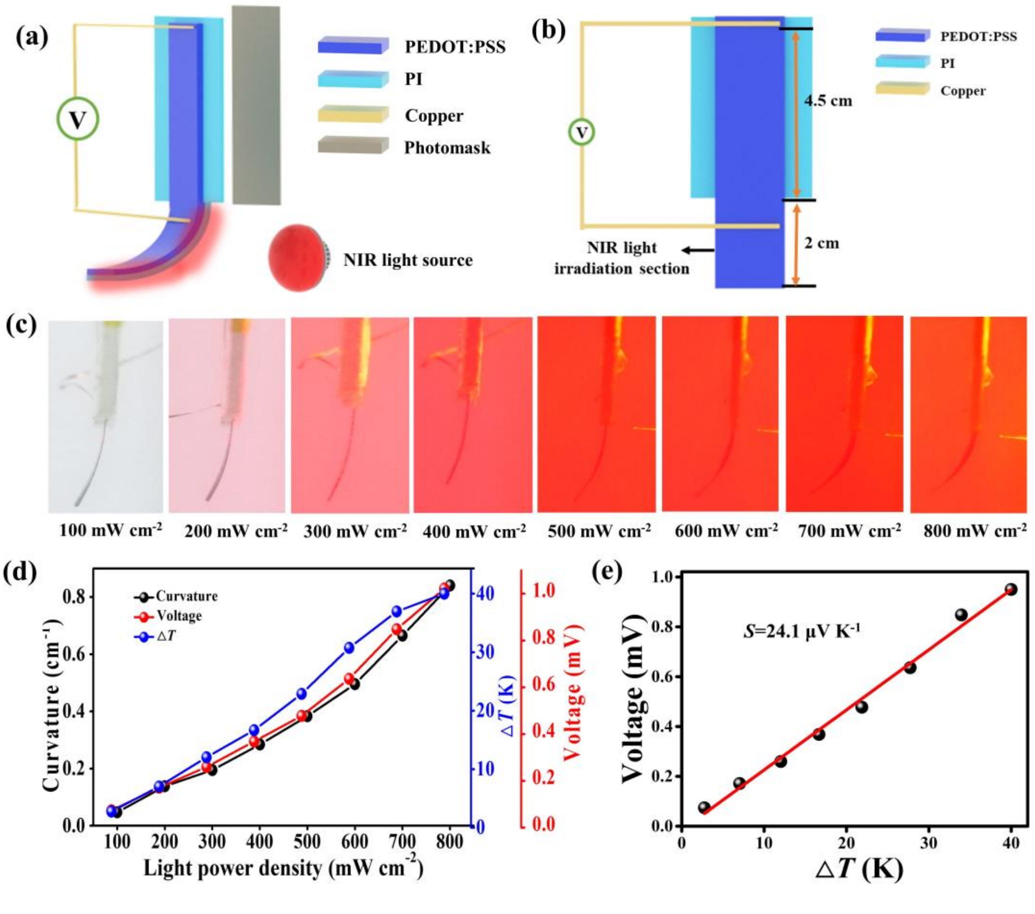

Fig. S20. (a) Schematic diagram of the actuation and PTE properties of the PEDOT:PSS/PI actuator. (b) Dimensions of the PEDOT:PSS/PI actuator. (c) Optical photographs of the shape deformation of the actuator under different light powers. (d) Bending curvature, Δ*T* and output voltage of the PEDOT:PSS/PI actuator as a function of light power density. (e) Seebeck coefficient of the PEDOT:PSS/PI actuator.

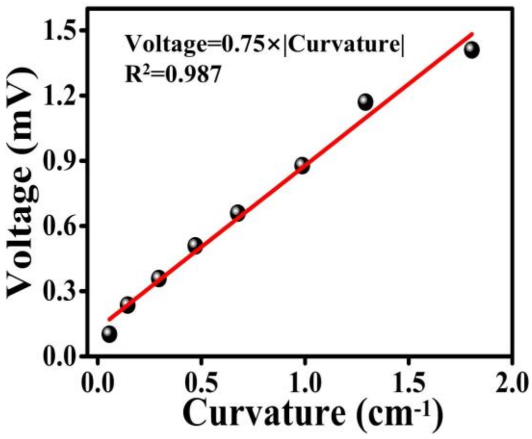

Fig. S21. Output voltage of PEDOT:PSS/MXene/PI actuator as a function of bending curvature.

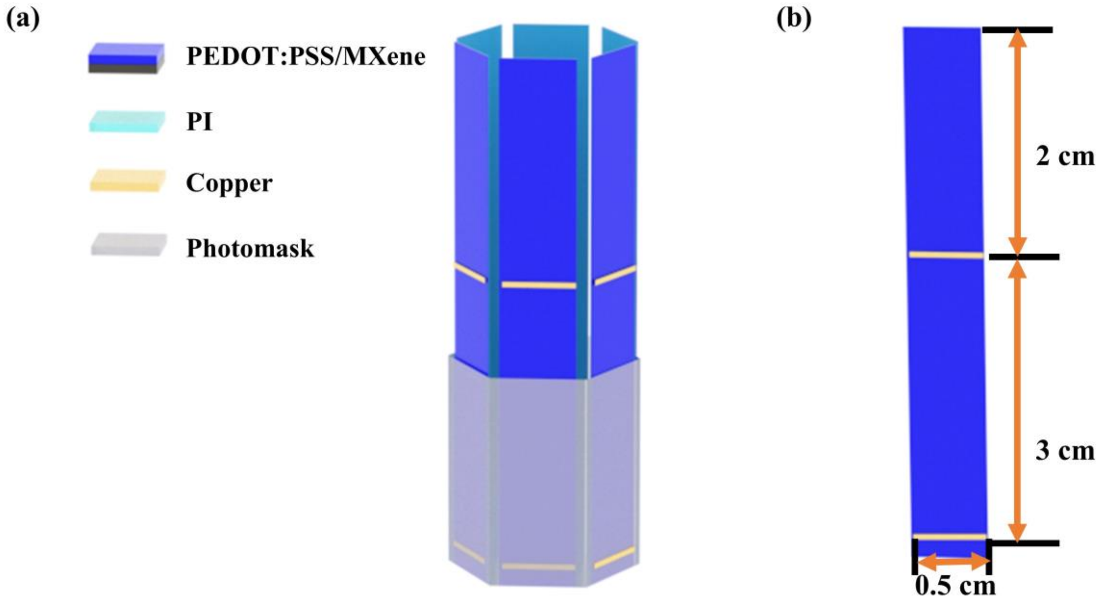

Fig. S22. (a) Schematic diagram of the bionic flower. (b) Dimensions of one PEDOT:PSS/MXene/PI actuator in the bionic flower.

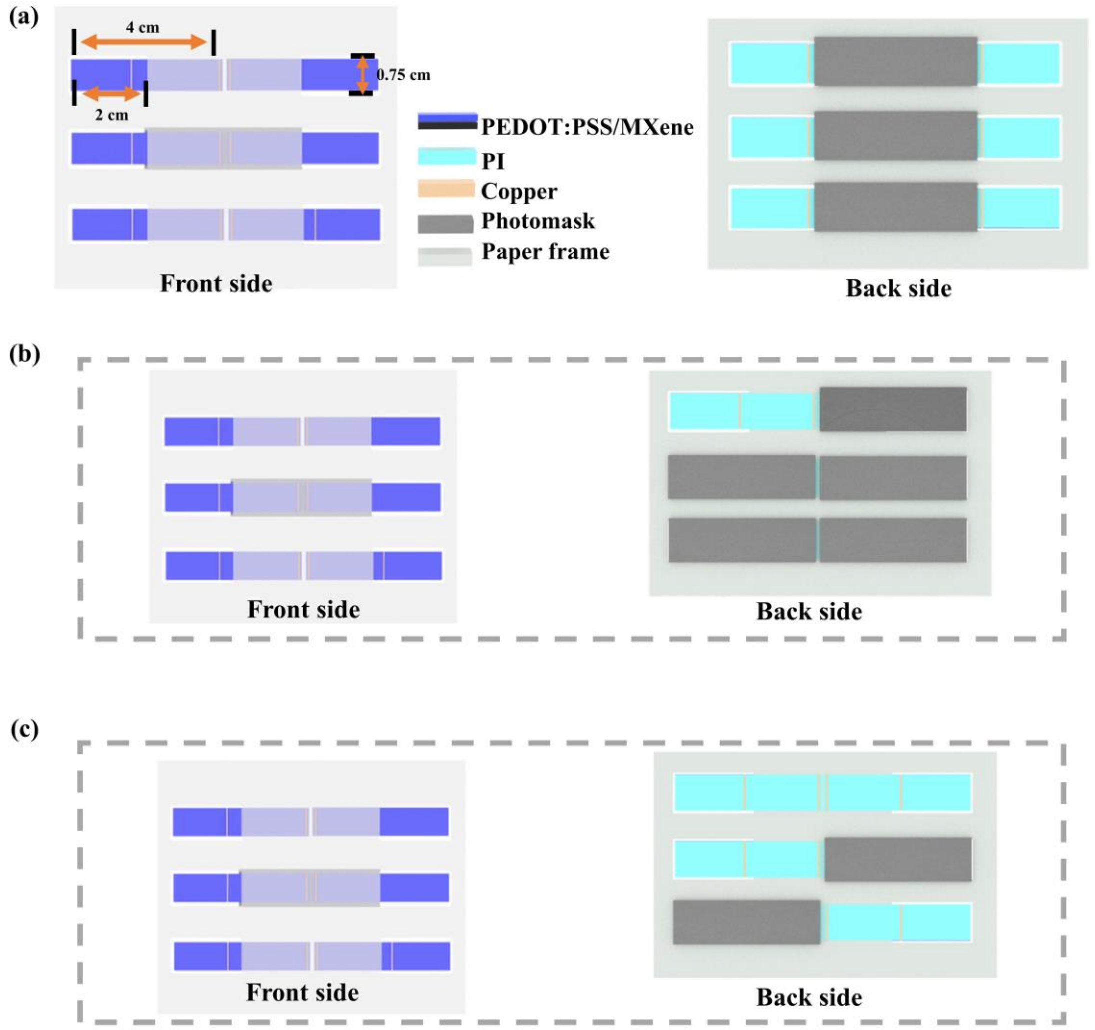

Fig. S23. (a) Dimensions and diagram of the front and back sides of the Braille device. (b) Schematic diagram of the front and back sides of Braille “a”. (c) Schematic diagram of the front and back sides of Braille “ed”.

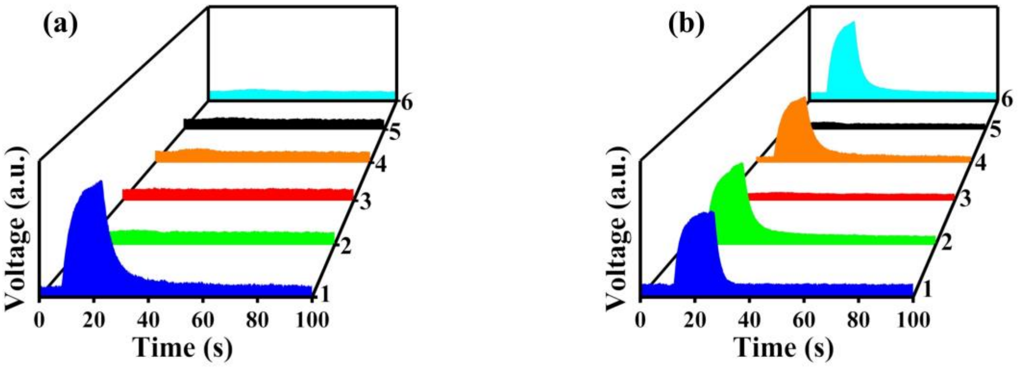

Fig. S24. (a) Real-time voltage signal variation for the Braille character “a”. (b) Real-time voltage signal variation for the Braille character “ed”.

Supplementary Note

Supplementary Note S1: Calculation of bending curvature of actuator.

In this experiment, these parameters of the actuator are defined as follows (shown in Fig. S17 (Supporting Information). *L* represents the length of the actuator. *X* represents the horizontal displacement of the actuator bending. *Y* represents the vertical displacement of the actuator bending. *r* represents the radius of the circle in which the arc actuator is located. θ represents the centre angle of the circle in which the arc actuator is located. *θ/2* represents the chord tangent angle of the circle. The reciprocal of the radius represents the curvature (*1/r*).

The chord tangent angle is obtained by the equation

$$\theta/2 = \tan^{-1}\frac{X}{L-Y} \qquad (1)$$

Here, the bending angle is defined by

$$\theta = \frac{L}{r} \qquad (2)$$

The curvature is derived as

$$\frac{1}{r} = \frac{\theta}{L} = \frac{2}{L}\tan\frac{X}{L-Y} \qquad (3)$$
